# β-Lapachone suppresses neuroinflammation by modulating the expression of cytokines and matrix metalloproteinases in activated microglia

**DOI:** 10.1186/s12974-015-0355-z

**Published:** 2015-07-16

**Authors:** Eun-Jung Lee, Hyun-Myung Ko, Yeon-Hui Jeong, Eun-Mi Park, Hee-Sun Kim

**Affiliations:** Department of Molecular Medicine, Tissue Injury Defense Research Center, School of Medicine, Ewha Womans University, Mok-6-dong 911-1, Yangchun-Ku, Seoul, 158-710 South Korea; Department of Pharmacology, Tissue Injury Defense Research Center, School of Medicine, Ewha Womans University, Seoul, 158-710 South Korea

**Keywords:** β-Lapachone, Microglia, Neuroinflammation, Cytokine, MMP, Signaling pathway

## Abstract

**Background:**

β-Lapachone (β-LAP) is a natural naphthoquinone compound isolated from the lapacho tree (*Tabebuia* sp.), and it has been used for treatment of rheumatoid arthritis, infection, and cancer. In the present study, we investigated whether β-LAP has anti-inflammatory effects under in vitro and in vivo neuroinflammatory conditions.

**Methods:**

The effects of β-LAP on the expression of inducible nitric oxide synthase (iNOS), cytokines, and matrix metalloproteinases (MMPs) were examined in lipopolysaccharide (LPS)-stimulated BV2 microglial cells and rat primary microglia by ELISA, reverse transcription polymerase chain reaction (RT-PCR), and Western blot analysis. Microglial activation and the expression levels of proinflammatory molecules were measured in the LPS-injected mouse brain by immunohistochemistry and RT-PCR analysis. The detailed molecular mechanism underlying the anti-inflammatory effects of β-LAP was analyzed by electrophoretic mobility shift assay, reporter gene assay, Western blot, and RT-PCR analysis.

**Results:**

β-LAP inhibited the expression of iNOS, proinflammatory cytokines, and MMPs (MMP-3, MMP-8, MMP-9) at mRNA and protein levels in LPS-stimulated microglia. On the other hand, β-LAP upregulated the expressions of anti-inflammatory molecules such as IL-10, heme oxygenase-1 (HO-1), and the tissue inhibitor of metalloproteinase-2 (TIMP-2). The anti-inflammatory effect of β-LAP was confirmed in an LPS-induced systemic inflammation mouse model. Thus, β-LAP inhibited microglial activation and the expressions of iNOS, proinflammatory cytokines, and MMPs in the LPS-injected mouse brain. Further mechanistic studies revealed that β-LAP exerts anti-inflammatory effects by inhibiting MAPKs, PI3K/AKT, and NF-κB/AP-1 signaling pathways in LPS-stimulated microglia. β-LAP also inhibited reactive oxygen species (ROS) production by suppressing the expression and/or phosphorylation of NADPH oxidase subunit proteins, such as p47^phox^ and gp91^phox^. The anti-oxidant effects of β-LAP appeared to be related with the increase of HO-1 and NQO1 via the Nrf2/anti-oxidant response element (ARE) pathway and/or the PKA pathway.

**Conclusions:**

The strong anti-inflammatory/anti-oxidant effects of β-LAP may provide preventive therapeutic potential for various neuroinflammatory disorders.

**Electronic supplementary material:**

The online version of this article (doi:10.1186/s12974-015-0355-z) contains supplementary material, which is available to authorized users.

## Background

Microglia are innate immune cells of the central nervous system that constantly move through the brain parenchyma and constitute an immune surveillance system [1, 2]. Microglia become activated in response to various stimuli or injury and produce inflammatory mediators such as nitric oxide (NO), cytokines, and matrix metalloproteinases (MMPs). Alternatively, activated microglia produce anti-inflammatory cytokines and lead to matrix deposition and wound healing [3, 4]. Thus, the balance between the inflammatory M1 and the anti-inflammatory M2 phase of microglial activation is important to maintain homeostasis in the brain. However, prolonged and unresolved inflammatory response leads to destructive, chronic inflammation (neuroinflammation) that results in neuronal cell death and ultimately in the onset of neurodegenerative diseases [5, 6]. Therefore, inhibition of exaggerated inflammatory responses by microglia has been suggested as an important strategy to develop therapeutic agents for various neuroinflammatory disorders.

β-Lapachone (3,4-dihydro-2,2-dimethyl-2*H*-naphtho[1,2-b]pyran-5,6-dione; β-LAP) is a natural compound which was originally isolated from the bark of the South American lapacho tree (*Tabebuia avellanedae*) [7]. β-LAP has been reported to have a wide variety of pharmacological effects including anti-inflammatory, anti-cancer, anti-bacterial, anti-fungal, anti-platelet, and anti-angiogenic action [8–10]. In particular, β-LAP exerts anti-neoplastic effects against various human cancer cell lines, and it is now being used in clinical trials for the treatment of various forms of cancer [11–13]. β-LAP has topoisomerase inhibitory activity and elevates NQO1 levels, leading to a futile redox cycle and apoptosis of cancer cells [11, 14]. A recent study reports that β-LAP attenuates cisplatin-mediated nephrotoxicity by increasing NAD^+^ levels with elevated tumoricidal effects of cisplatin [15]. Several studies have reported anti-inflammatory effects of β-LAP. β-LAP suppresses inflammatory responses in activated macrophages and protects from lung edema and high mortality in septic mice [16]. β-LAP alleviates carrageenan-induced rat paw edema by suppressing neutrophil migration and cytokine production [17]. In addition, β-LAP induces anti-inflammatory heme oxygenase-1 (HO-1) via AMPK activation in RAW264.7 macrophages and endothelial cells [18, 19]. A previous study has shown anti-inflammatory effects of β-LAP in activated microglia [20]. It was demonstrated that β-LAP inhibits inducible nitric oxide synthase (iNOS) and cytokine expressions in lipopolysaccharide (LPS)-stimulated BV2 cells. However, the in vivo effects of β-LAP and the detailed molecular mechanism underlying the anti-inflammatory effects of β-LAP have not been fully elucidated.

Therefore, in the present study, we examined the anti-inflammatory effects of β-LAP under both in vitro and in vivo neuroinflammatory conditions and analyzed, in detail, the molecular mechanism. In particular, we investigated the effects of β-LAP on the gene expression and activity of MMPs, because our group recently demonstrated the proinflammatory role of MMPs in activated microglia [21–23]. Through this study, we report for the first time that β-LAP inhibits microglial activation and expression of iNOS, cytokines, and several MMPs in the LPS-injected mouse brain. Furthermore, we demonstrated that multiple signaling pathways are involved in the anti-inflammatory mechanism of β-LAP in activated microglia.

## Materials and methods

### Reagents and antibodies

All reagents for cell culture were purchased from Gibco BRL (Grand Island, NY, USA). β-Lapachone and LPS (*Escherichia coli* serotype 055:B5) were obtained from Sigma–Aldrich (St. Louis, MO, USA). All reagents and enzymes for reverse transcription polymerase chain reaction (RT-PCR) and oligonucleotides for electrophoretic mobility shift assay (EMSA) were purchased from Promega (Madison, WI, USA). Antibodies against phospho-/total forms of MAPKs, CREB, β-actin, MMPs (MMP-3, MMP-8, MMP-9), and tissue inhibitor of metalloproteinase-2 (TIMP-2) were supplied by Cell Signaling Technology (Beverley, CA, USA), Abcam (Cambridge, UK), or Chemicon (Temecula, CA, USA). Antibodies against HO-1, NQO1, and Iba1 were purchased from Santa Cruz Biotechnology (Santa Cruz, CA, USA) or Novus (Littleton, CO, USA). The antibody for phospho-p47^phox^ (Ser370) was purchased from Assay Biotechnology Company Inc. (Sunnyvale, CA, USA). All other chemicals were obtained from Sigma–Aldrich, unless otherwise stated.

### Microglial cell cultures

The immortalized mouse BV2 microglial cell line [24] was grown and maintained in Dulbecco’s modified Eagle’s medium (DMEM), supplemented with 10 % heat-inactivated fetal bovine serum, streptomycin (10 μg/ml), and penicillin (10 U/ml) at 37 °C under 5 % CO_2_. Primary microglial cells were cultured from the cerebral cortices of 1- to 2-day-old Sprague Dawley rat pups as described previously [21]. The purity of microglial cultures was >95 %, as confirmed by Western blot and immunocytochemistry analyses using an antibody specific to ionized calcium-binding adapter protein-1 (IBA-1) staining (data not shown).

### Measurement of cytokines, nitrite, and intracellular ROS levels

Cells (1 × 10^5^ cells per well in a 48-well plate) were pretreated with β-LAP for 1 h and further stimulated with LPS (100 ng/ml) for 16 h. Concentrations of TNF-α, IL-1β, IL-6, and IL-10 in conditioned medium (CM) were measured by ELISA using monoclonal antibodies and procedures recommended by the supplier (PharMingen, San Diego, CA). Accumulated nitrite in CM and intracellular accumulation of reactive oxygen species (ROS) were measured using Griess reagent (Promega) and H_2_DCF-DA (Invitrogen, La Jolla, USA), respectively, as previously described [25].

### Assays for MMP-3, MMP-8, and MMP-9 activity

BV2 cells were stimulated with LPS in the presence or absence of β-LAP for 24 h, and the supernatants were collected to measure MMP activity using the SensoLyte® 520 MMP assay system (AnaSpec, San Jose, CA, USA). MMP activity measurements were performed by continuous detection of peptide cleavage using a fluorescence plate reader (Molecular Devices, Sunnyvale, CA, USA). MMP activity units were expressed as a change in the fluorescence intensity at an excitation wavelength of 490 nm and an emission wavelength of 520 nm.

### LPS-induced inflammation and administration of β-LAP

C57BL/6 mice (10–11 weeks old) were purchased from the Orient Co., Ltd. (Seoul, Korea). All animal experiments were approved by the Institutional Animal Care and Use Committee at the School of Medicine, Ewha Womans University. All efforts were made to minimize animal suffering, to reduce the number of animals used, and to utilize alternatives to in vivo techniques, if available. Systemic inflammation was induced by LPS administration (5 mg/kg, i.p.) to male C57BL/6 mice as previously described [26]. β-LAP (10 mg/kg, i.p.), dissolved in vehicle solution (1 % DMSO and normal saline), was given daily for 4 days before the LPS treatment. Samples were obtained 3 or 6 h after LPS treatment.

### Immunohistochemistry

Three hours after LPS treatment, the animals were anesthetized with sodium pentobarbital (120 mg/kg i.p.) and perfused transcardially with normal saline containing heparin (5 U/ml), followed by 4 % paraformaldehyde (PFA) in 0.1 M sodium phosphate buffer (PBS), pH 7.2. The brains were removed and incubated overnight in fixatives and stored in a 30 % sucrose solution. Serial coronal brain sections of regions containing the hippocampus (20 μm thick, at 600-μm intervals) were collected using a cryostat. Brain sections were incubated in PBS containing 0.1 % Triton X-100, 5 % normal serum, and 1 % bovine serum albumin for 1 h, and then subsequently incubated with primary antibody. On the next day, sections were incubated in a 1:200 dilution of Alexa Fluor 488-labeled donkey anti-rabbit secondary antibody or Alexa Fluor 594-labeled chicken anti-goat antibody (Molecular Probes Inc., Eugene, OR, USA) for 60 min at room temperature, and then washed with 0.05 % Tween 20 in PBS three times, 5 min each. Sections were then stained with a 0.5-μg/ml DAPI staining solution for 20 min at room temperature and washed. The sections were mounted with Vectashield mounting medium (Vector Laboratories, Burlingame, CA, USA), and fluorescence microcopy images were obtained using confocal microscopy (TSC-SP, Leica, Heidelberg, Germany). Iba1-, MMP-3-, MMP-8-, and MMP-9-positive cells were quantified using the Metamorph program (Carl Zeiss, Jena, Germany). Two serial brain sections from each animal were used for further analysis, and quantification of Iba1-, MMP-3-, MMP-8-, and MMP-9-positive cells was performed in three different areas (500 μm^2^ in size) in the lateral cortex and dentate gyrus of the right hemisphere per brain section. The mean cell number from six 500-μm^2^ areas per animal was calculated.

### RT-PCR

Total RNA (1 μg) isolated from BV2 or primary microglial cells (4.5 × 10^5^ cells on a 6-cm dish), or from the brain tissue of LPS-injected mice, was reverse transcribed, and synthesized cDNA was used as a template for PCR. RT-PCR was performed on a T100 Thermal Cycler (Bio-Rad) with GoTaq polymerase (Promega). The primer sets shown in Table 1 were used to detect specific PCR products, and their values were calculated as fold change relative to control after normalization to the GAPDH gene.

**Table 1:** Primers used in RT-PCR reactions

Species	Gene	Forward primer (5′ → 3′)	Reverse primer (5′ → 3′)	Size (bp)
Mouse	TNF-α	CCTATGTCTCAGCCTCTTCT	CCTGGTATGAGATAGCAAAT	354
	iNOS	CAAGAGTTTGACCAGAGGACC	TGGAACCACTCGTACTTGGGA	450
	IL-1β	GGCAACTGTTCCTGAACTCAACTG	CCATTGAGGTGGAGAGCTTTCAGC	447
	IL-6	CCACTTCACAAGTCGGAGGCTT	CCAGCTTATCTGTTAGGAGA	395
	IL-10	GCCAGTACAGCCGGGAAGACAATA	GCCTTGTAGACACCTTGGTCTT	409
	MMP-3	ATTCAGTCCCTCTATGGA	CTCCAGTATTTGTCCTCTAC	375
	MMP-8	CCAAGGAGTGTCCAAGCCAT	CCTGCAGGAAAACTGCATCG	180
	MMP-9	GTGATCCCCACTTACTATGGAAAC	GAAGCCATACAGTTTATCCTGGTC	352
	TIMP-2	TCTAATTGCAGGAAAGGCAGA	TGCTCTTCTCTGTGACCCAGT	218
	HO-1	TGTCACCCTGTGCTTGACCT	ATACCCGCTACCTGGGTGAC	209
	NQO1	AGAGGCTCTGAAGAAGAGAGG	CACCCTGAAGAGAGTACATGG	401
	p47^phox^	CGATGGATTGTCCTTTGTGC	ATCACCGGCTATTTCCCATC	256
	p67^phox^	CTTCAACATAGGCTGCGTGA	CTTCATGTTGGTTGCCAATG	334
	p22^phox^	AAAGAGGAAAAAGGGGTCCA	TAGGCTCAATGGGAGTCCAC	239
	gp91^phox^	GTCAAGTGCCCCAAGGTATCCA	TTGTAGCTGAGGAAGTTGGC	453
	GAPDH	ATGTACGTAGCCATCCAGGC	AGGAAGGAAGGCTGGAAGAG	420
Rat	TNF-α	AAGTTCCCAAATGGGCTCCCT	TGAAGTGGCAAATCGGCTGAC	306
	iNOS	GCAGAATGTGACCATCATGG	ACAACCTTGGTGTTGAAGGC	426
	IL-1β	AAATGCCTCGTGCTGTCTGACC	TCCCGACCATTGCTGTTTCCT	377
	IL-6	TCATTCTGTCTCGAGCCCAC	GAAGTAGGGAAGGCAGTGGC	345
	IL-10	AGGGCTGCCTTCAGTCAAGT	AGAAATCGATGACAGCGTCG	396
	MMP-3	GTACCAACCTATTCCTGGTTGC	CCAGAGAGTTAGATTTGGTGGG	231
	MMP-8	TACAACCTGTTTCTCGTGGCTGC	TCAACTGTTCTCAGCTGGGGATG	317
	MMP-9	AAGTTGAACTCAGCCTTTGAGG	GTCGAATTTCCAGATACGTTCC	225
	TIMP-2	CGTAGTGATCAGAGCCAAGC	TCTGCCTTTCCTGCAATTAGA	225
	GAPDH	GTGCTGAGTATGTCGTGGAGTCT	ACAGTCTTCTGAGTGGCAGTGA	292

### Western blot analysis

Proteins isolated from total cell lysates, and from CM, were separated by SDS-PAGE, transferred to nitrocellulose membranes, and incubated with primary antibodies against MMP-3, MMP-8, and MMP-9; TIMP-2 (1:1000); the phospho- or total form of MAP kinases or CREB; HO-1; NQO1 (1:1000); or p-p47^phox^ [anti-phospho-(Ser345)-p47phox Ab] (1:1000, Assay Biotechnology). After thorough washing with Tris-buffered saline with Tween 20 (TBST), horseradish peroxidase-conjugated secondary antibodies (1:2000 dilution in TBST; New England Biolabs, Beverly, MA, USA) were applied, and the blots were developed using an enhanced chemiluminescence detection kit (Pierce Biotechnology, Rockford, IL, USA). To detect secreted MMPs, MMP proteins in the conditioned media were enriched using an Amicon® centrifugal filter (Millipore Corp., Billerica, MA, USA).

### Transient transfection and luciferase assay

BV2 cells plated at 50–60 % confluence (2 × 10^5^ cells per well) in 12-well plates were transfected with 1 μg of plasmid DNA (ARE-luc, CRE-luc) using the Convoy™ Platinum transfection reagent (CellTAGen, Seoul, Korea). After 36 h of transfection, cells were treated with β-LAP and LPS (100 ng/ml), or β-LAP only, for 6 h. The luciferase assay was used to determine the effect of β-LAP on ARE or CRE promoter activity. The ARE-luciferase reporter gene was kindly provided by Dr. Young-Joon Surh (Seoul National University, Seoul, Korea). The sequence of the anti-oxidant response element (ARE) construct is as follows: 5′-CTCAGCCTTCCAAATCG CAGTCACAGTGACTCAGCAGAATC-3′ [27, 28]. The CRE-luc vector, which contains four copies of the cyclic AMP response element (CRE, TGACGTCA), was obtained from Stratagene (La Jolla, CA).

### EMSA

Nuclear extracts from treated microglia were prepared as follows. Cells (2 × 10^7^) were treated with 1 ml of lysis buffer (10 mM Tris–HCl, pH 7.9; 10 mM NaCl; 3 mM MgCl_2_; 1 % NP-40) on ice for 5 min. After 10 min of centrifugation at 3000 rpm, the pellet was resuspended in 50 μl of extraction buffer (20 mM HEPES, pH 7.9; 20 % glycerol; 1.5 mM MgCl_2_; 0.2 mM EDTA; 300 mM NaCl; 1 mM DTT; 1 mM PMSF) and incubated on ice for 30 min. After centrifugation at 13,200 rpm for 15 min, the supernatant was harvested as a nuclear protein extract and stored at −70 °C. Double-stranded DNA oligonucleotides containing the NF-κB, AP-1, ARE, or CRE consensus sequences were end labeled using T4 polynucleotide kinase (New England Biolabs, Beverly, MA) in the presence of [γ-^32^P]ATP. Nuclear proteins (5 μg) were incubated with ^32^P-labeled probe on ice for 30 min, resolved on a 5 % acrylamide gel, and visualized by autoradiography. We purchased double-stranded DNA oligonucleotides containing the NF-κB, AP-1, or CRE consensus sequences from Promega (Madison, WI, USA) and that containing ARE from Santa Cruz Biotechnology (Santa Cruz, CA, USA). The DNA sequences of the probes are as follows: NF-κB (5′-AGTTGAGGGGAC TTTCCCAGGC-3′), AP-1 (5′-CGCTTGATGAGTCAGCCGGAA-3′), ARE (5′-TGG GGAACCTGTGCTGAGTCACTGGAG-3′), and CRE (5′-AGAGATTGCCTGACGTCAGAGAGCTA-3′).

### Statistical analysis

Unless otherwise stated, all experiments were performed with triplicate samples and repeated at least three times. Data are presented as mean ± SEM, and statistical comparisons among groups were performed using one-way ANOVA followed by Newman–Keuls post hoc tests or *t* tests. Statistical significance was accepted for *P* values <0.05.

## Results

### β-LAP showed anti-inflammatory effects in LPS-stimulated microglial cells

**Fig. 1:**
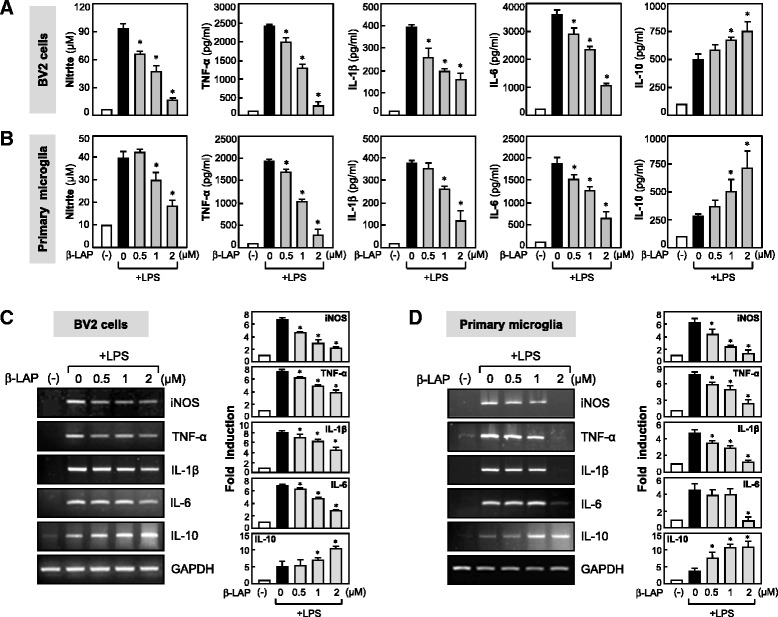
Effects of β-LAP on iNOS and pro-/anti-inflammatory cytokines in LPS-stimulated BV2 cells and primary cultured microglia. BV2 cells (**a**) or primary cultured microglia (**b**) were pretreated with β-LAP (0.5, 1, and 2 μM) for 1 h and incubated with LPS (100 ng/ml for BV2, 10 ng/ml for primary microglia). After incubation for 16 h, the conditioned media were collected, and the amounts of NO, TNF-α, IL-1β, IL-6, and IL-10 were measured using Griess reagent or ELISA. The data are the mean ± SEM of three independent experiments. *P < 0.05, significantly different from LPS-treated samples. BV2 cells (**c**) or primary microglia (**d**) were pretreated with β-LAP (0.5, 1, and 2 μM) for 1 h, followed by LPS (100 ng/ml) for 6 h, and total RNA was isolated. The mRNA expressions of iNOS and cytokines were analyzed by RT-PCR. Representative gels are shown on the *left panel*, and quantifications of three independent experiments are shown in the *right panel*. Values correspond to the mean ± SEM of three independent experiments. *P < 0.05, significantly different from LPS-treated samples

To investigate the anti-inflammatory effect of β-LAP, BV2 or primary microglial cells were treated with LPS in the presence or absence of β-LAP for 16 h. Consecutively, NO and cytokine levels in CM were measured. We observed that β-LAP significantly inhibited the LPS-induced production of NO and proinflammatory cytokines such as TNF-α, IL-1β, and IL-6, whereas it raised anti-inflammatory IL-10 in BV2 cells and primary microglia (Fig. 1a, b). β-LAP did not have any cytotoxicity in the concentrations used in this study in both the BV2 and primary microglial cells, at least for 48 h (MTT assay, data not shown). Subsequent RT-PCR analysis showed that β-LAP reduced the mRNA expression of iNOS, TNF-α, IL-1β, and IL-6, and increased IL-10 (Fig. 1c, d). The data suggest that β-LAP modulates the expressions of iNOS and cytokines at the transcriptional level.

### β-LAP suppressed the expression and activity of MMP-3, MMP-8, and MMP-9, while it enhanced TIMP-2 expression in LPS-stimulated microglia

**Fig. 2:**
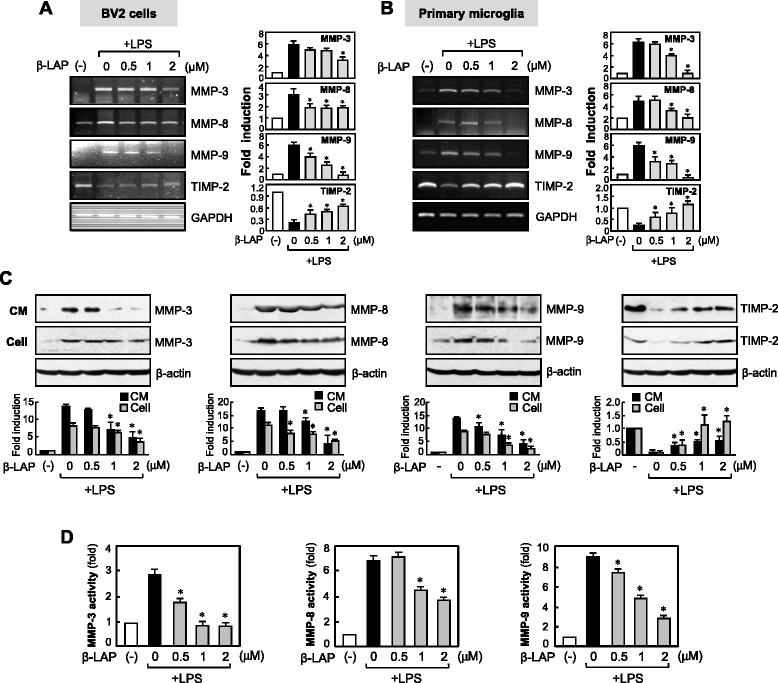
β-LAP suppressed the LPS-induced expression and enzymatic activity of MMP-3, MMP-8, and MMP-9, whereas it enhanced TIMP-2 expression. BV2 cells (**a**) or primary microglia (**b**) were pretreated with β-LAP (0.5, 1, and 2 μM, for 1 h), followed by LPS (100 or 10 ng/ml), and total RNA was isolated at 6 h after LPS treatments. The mRNA expressions of MMPs and TIMP-2 were analyzed by RT-PCR. Representative gels are shown in the *left panel*, and quantification data are shown in the *right panel* (*n* = 3). **c** Western blot analysis was performed using conditioned medium (CM) or cell lysates of BV2 cells pretreated with β-LAP (0.5, 1, and 2 μM, for 1 h), followed by LPS (100 ng/ml) for 16 h. Levels of MMP-3, MMP-8, and MMP-9, and TIMP-2 protein expression were normalized using β-actin and were expressed as relative fold changes in comparison with control samples. **d** The enzymatic activities of MMPs in the CM were detected using MMP activity assay kits. BV2 cells were pretreated with β-LAP (0.5, 1, and 2 μM, for 1 h), followed by LPS (100 ng/ml, for 24 h), and the CM was collected to measure MMP activity. MMP activity units were expressed as a change in fluorescence intensity. Values are expressed as the means ± SEM for three independent experiments. *P < 0.05, significantly different from the LPS-treated group

Our group recently reported that MMP-3, MMP-8, and MMP-9 are important proinflammatory mediators in activated microglia, while TIMP-2 plays an anti-inflammatory role [21–23, 29]. To investigate whether β-LAP affects the expression of MMPs and TIMP-2, we performed RT-PCR analysis and Western blotting using cell lysates and CM from LPS + β-LAP-treated microglial cells. We found that β-LAP inhibited LPS-induced expressions of MMP-3, MMP-8, and MMP-9 at the mRNA and protein levels in both BV2 cells and primary microglia (Fig. 2a, b). Moreover, β-LAP reduced the enzymatic activity of MMP-3, MMP-8, and MMP-9 in the CM (Fig. 2d). On the other hand, β-LAP restored the expression of TIMP-2 (Fig. 2a–c). The results suggest that the inhibition of MMPs and the upregulation of TIMP-2 are at least partly involved in the anti-inflammatory effects of β-LAP.

### β-LAP inhibited microglial activation and the expression of iNOS, cytokines, and MMPs in the brains of LPS-induced systemic inflammation mice

**Fig. 3:**
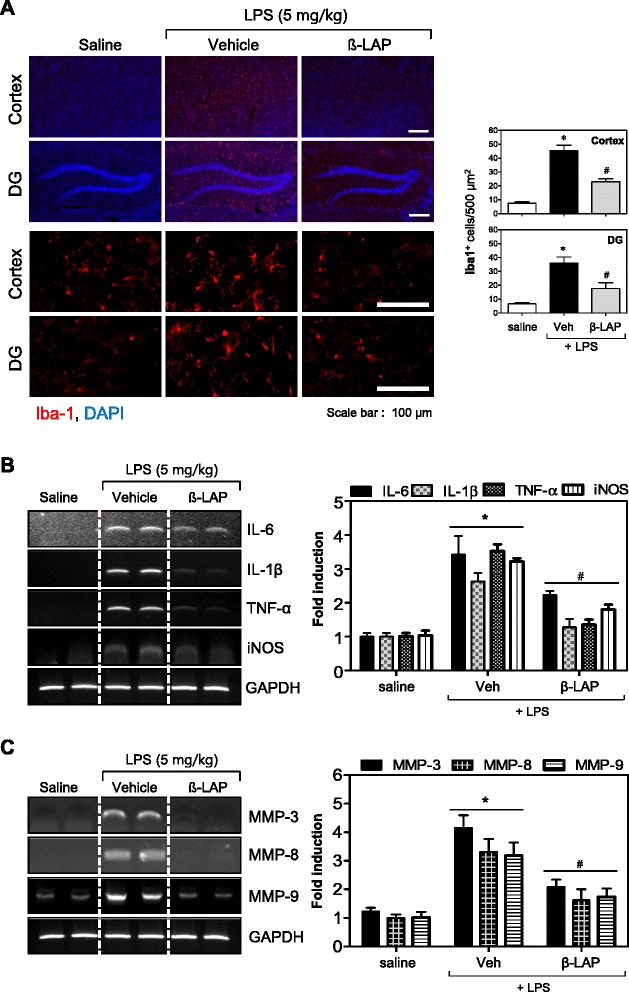
β-LAP reduced neuroinflammation induced by systemic LPS administration. **a** Immunofluorescence labeling of Iba1 (*red*) and quantification of the number of activated Iba1-positive cells 3 h after systemic LPS treatment (5 mg/kg, i.p.). Nuclei are counterstained with DAPI (*blue*). Microglial activation in the cortex and dentate gyrus (DG) of LPS-injected mouse was reduced by β-LAP (10 mg/kg, i.p., daily for 4 days) treatment. Representative images were obtained from one set of experiments, and the three experiments were performed independently. *Upper images* are the results of Iba1 + DAPI staining with an original magnification of ×40. *Lower images* are the results of Iba1 staining with an original magnification of ×400. **b**, **c** β-LAP reduced the mRNA expression of proinflammatory cytokines (IL-6, IL-1β, TNF-α), iNOS, and MMPs (MMP-3, MMP-8, and MMP-9) in the cortex of LPS-injected mice (5 mg/kg, 3 h). Representative gels are shown in the *left panel*, and quantification data are shown in the *right panel* (*n* = 3 in each group). Results are representative RT-PCR data in the cortex 3 h after LPS treatment. Values are expressed as the means ± SEM for three independent experiments. *P < 0.05 vs. saline or ^#^ *P* < 0.005 vs. LPS-treated mice

**Fig. 4:**
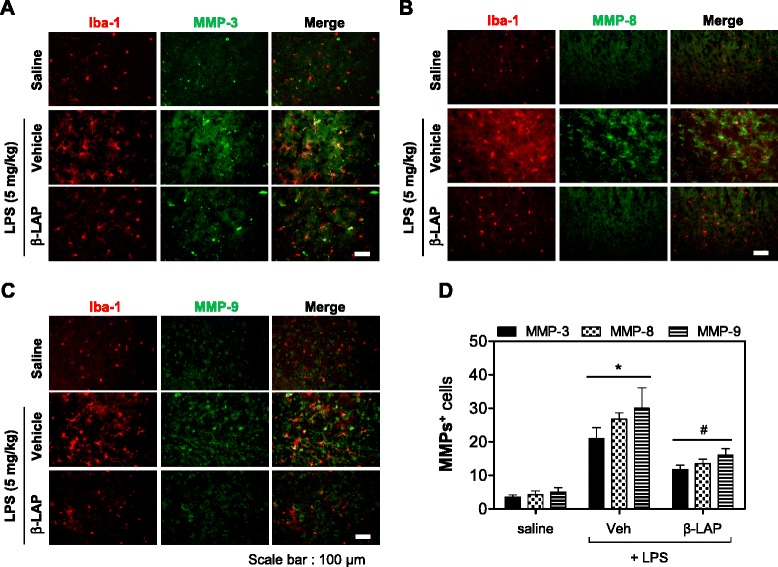
β-LAP suppressed MMP-3, MMP-8, and MMP-9 expression in the LPS-induced systemic inflammation mouse brain. Changes in protein expression of MMP-3 (**a**), MMP-8 (**b**), and MMP-9 (**c**) were determined in LPS (24 h)-injected mouse brains. The number of activated Iba1^+^ cells with thick and densely stained processes was markedly increased in the cortex at 24 h after systemic LPS treatment (5 mg/kg, i.p.), compared to saline groups. MMP-3, MMP-8, and MMP-9 expression in the cortex of LPS-injected mouse was reduced by treatment with β-LAP (10 mg/kg, i.p., daily for 4 days). Representative images of MMP-positive cells and double-positive cells (MMP, *green*; Iba-1, *red*), as determined by immunohistochemistry. Representative images (**a**–**c**) and quantification of the data (**d**). Values represent the number of double-immunopositive cells. *Scale bar*, 100 μm. *n* = 3 per group. *P < 0.05 vs. saline or ^#^ *P* < 0.005 vs. LPS-treated mice

To verify the anti-inflammatory effects of β-LAP in vivo, β-LAP (10 mg/kg) was injected before LPS administration. After 3 h, microglial activation and the expression levels of proinflammatory cytokines and MMPs were measured in the LPS-injected mouse brain. Systemic LPS led to an increase in densely stained Iba1-positive cells in the cortex and dentate gyrus (Fig. 3a). This indicates the presence of activated microglia. Pretreatment of β-LAP, however, reduced their number. The smaller amount of densely stained activated microglia was significant as seen in the subsequent quantification of Iba1-positive cells. However, we did not observe any infiltration of neutrophils or monocytes into the brain at this time point (i.e., 3 h) (data not shown). In addition, we found that β-LAP inhibited the mRNA expression of proinflammatory cytokines (IL-6, IL-lβ, TNF-α), iNOS, and MMPs (MMP-3, MMP-8, MMP-9) that were elevated in the cortex region of the LPS-injected mouse brain (Fig. 3b, c). To determine whether MMPs, upregulated in the cortex of LPS-injected mouse, were co-localized with microglia, immunohistochemistry was performed using antibodies against Iba1 (red) and MMP-3, MMP-8, or MMP-9 (green), 24 h after the LPS challenge. The number of double-immunopositive cells was significantly increased by LPS injection, but it was reduced in the β-LAP-injected groups (Fig. 4). The results indicate that β-LAP inhibits the expression of MMP-3, MMP-8, and MMP-9 in the microglia of the LPS-injected mouse brain.

### β-LAP inhibited the phosphorylation of MAPKs and AKT and the DNA binding activity of NF-κB and AP-1 in LPS-stimulated BV2 cells

**Fig. 5:**
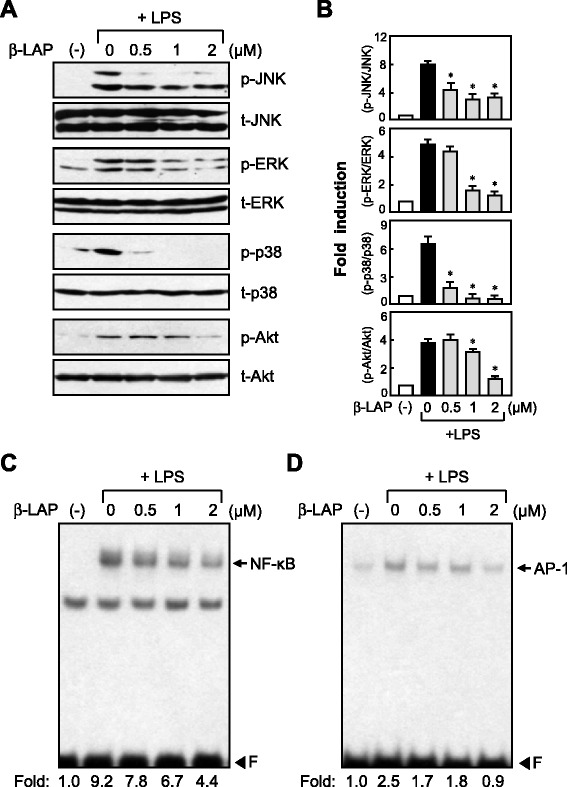
β-LAP inhibited the phosphorylation of MAPKs and AKT and DNA binding of NF-κB and AP-1 in LPS-stimulated BV2 cells. **a** Western blots for MAPKs and AKT activities. Cell extracts were prepared from BV2 cells pretreated with β-LAP (0.5, 1, and 2 μM, for 1 h), followed by LPS (100 ng/ml, for 1 h), and then subjected to immunoblot analysis using antibodies against the phospho- or total forms of JNK, ERK, p38 MAPK, and Akt. The autoradiograms are representative of three independent experiments. **b** Quantification of Western blot data. Levels of the phosphorylated forms of MAPKs and AKT were normalized with respect to the level of each total form and expressed as relative fold changes vs. the control group. Data are the means ± SEM for three independent experiments. *P < 0.05, significantly different from the LPS-treated samples. **c**, **d** EMSA for NF-κB and AP-1 DNA binding activity. BV2 cells were pretreated with β-LAP (0.5, 1, and 2 μM, for 1 h), followed by LPS (100 ng/ml, for 3 h), and nuclear extracts prepared from BV2 cells were incubated with the NF-κB (**c**) or AP-1 (**d**) probes. The *arrow* indicates a DNA–protein complex of NF-κB or AP-1. *F* indicates a free probe

To further investigate the anti-inflammatory mechanism of β-LAP, we examined the effects of β-LAP on MAPKs and PI3K/AKT signaling, which play a critical role in the process of microglial activation and the production of NO and cytokines [30, 31]. We found that β-LAP significantly inhibited the phosphorylation of three types of MAPKs and AKT in LPS-stimulated BV2 cells (Fig. 5a, b). Furthermore, we found that β-LAP suppressed the DNA binding activity of NF-κB and AP-1, which are key transcription factors modulating cytokine and iNOS gene expression in microglia (Fig. 5c, d).

### β-LAP suppressed ROS production and expression of NADPH oxidase subunits, whereas it enhanced anti-oxidant enzyme HO-1/NQO1 expression

**Fig. 6:**
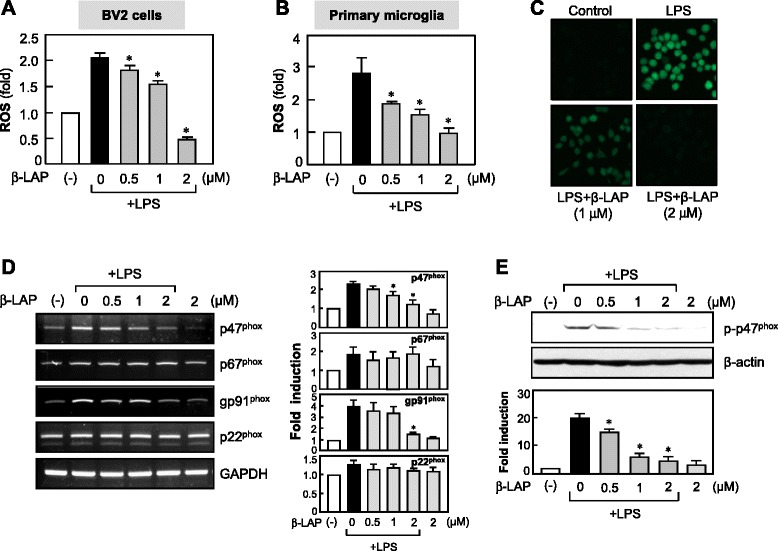
β-LAP inhibited ROS production via suppression of NADPH oxidase subunits. **a** BV2 cells or **b** primary microglia were pretreated with β-LAP (0.5, 1, and 2 μM, for 1 h), followed by LPS (100 or 10 ng/ml, for 16 h), and stained with 50 μM H_2_DCF-DA. DCF fluorescence intensities were measured using a microplate fluorometer. The data are expressed as the means ± SEM of three independent experiments. *P < 0.05, significantly different from the LPS-treated group. **c** A representative confocal image of DCF-derived fluorescence (*green*) in BV2 cells (*n* = 3), with an original magnification of ×200. **d** RT-PCR analysis for NADPH oxidase subunits (p47phox, p67phox, gp91phox, p22phox). BV2 cells were pretreated with β-LAP (0.5, 1, and 2 μM, for 1 h) followed by LPS (100 ng/ml), and total RNA was isolated at 2 h after LPS treatment. Representative gels are shown in the *left panel*, and quantification data are shown in the *right panel* (*n* = 3). **e** Western blot analysis for phosphorylation of the p47phox subunit (*n* = 3). BV2 cells were pretreated with β-LAP (0.5, 1, and 2 μM, for 1 h), followed by LPS (100 ng/ml, for 30 min), and then subjected to immunoblot analysis using antibodies against phospho-p47phox. Quantification data are shown in the graph. *P < 0.05, significantly different from the LPS-treated group

**Fig. 7:**
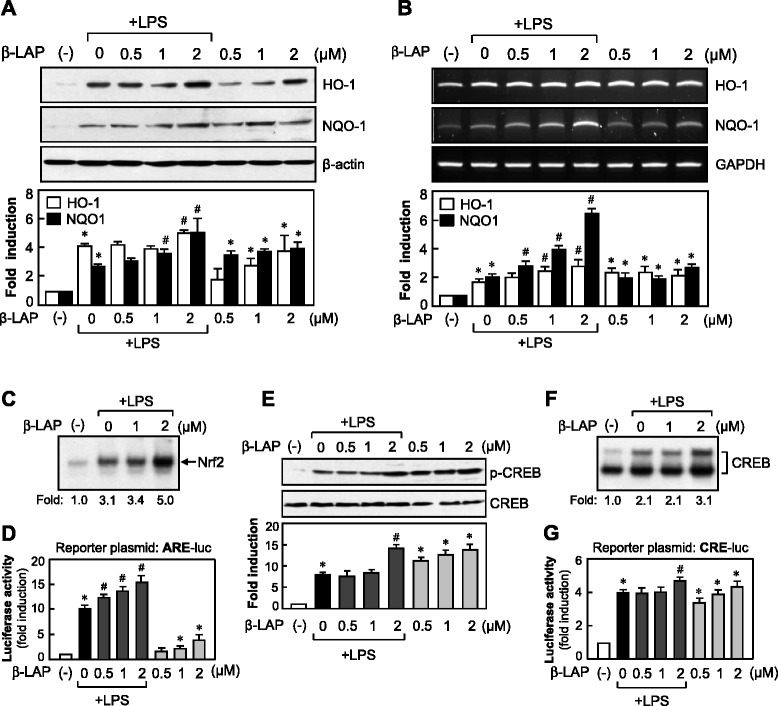
β-LAP increased HO-1 and NQO1 expression via upregulation of Nrf2/ARE and PKA/CREB pathways in LPS-stimulated microglia. **a** Western blot analysis shows the effects of β-LAP on HO-1 and NQO1 protein expression. Cell lysates were obtained from BV2 cells treated with β-LAP (0.5, 1, and 2 μM) with or without LPS (100 ng/ml) for HO-1 or NQO1 (6–16 h). **b** RT-PCR was performed to determine the HO-1 and NQO1 mRNA expression. Cells were treated with β-LAP (0.5, 1, and 2 μM) for 1 h prior to treatment with LPS (100 ng/ml, for 6 h) and analyzed. Quantification data are shown in the graph (*n* = 3). **c** EMSA for Nrf2. Nuclear extracts were prepared from BV2 cells treated with LPS (100 ng/ml, for 3 h) or LPS + β-LAP (1 and 2 μM, pretreatment for 1 h) and incubated with the ARE probe. The *arrow* indicates a DNA–protein complex of Nrf2. **d** Effect of β-LAP on ARE-luc reporter gene activity. Cells transfected with the reporter plasmid (ARE-luc) were treated with β-LAP (0.5, 1, and 2 μM) with or without LPS (100 ng/ml) for 6 h, and the reporter gene assay was performed. **e** Effect of β-LAP on the phosphorylation of CREB. Cell lysates were obtained from BV2 cells treated with β-LAP (0.5, 1, and 2 μM) with or without LPS (100 ng/ml) for 1 h. Quantification data are shown in the graph (*n* = 3). **f** EMSA for CREB. Nuclear extracts were prepared from BV2 cells treated with LPS (100 ng/ml, for 3 h) or LPS + β-LAP (1 and 2 μM, pretreatment for 1 h) and incubated with the CRE probe. The *bracket* indicates a DNA–protein complex of CREB. **g** Effect of β-LAP on CRE-luc activity. Cells transfected with the reporter plasmid (CRE-luc) were treated with β-LAP (0.5, 1, and 2 μM), with or without LPS (100 ng/ml) for 6 h, and the reporter gene assay was performed. Data are the means ± SEM of three independent experiments. *P < 0.05 vs. control or ^#^ *P* < 0.05 vs. the LPS-treated group

Next, we examined the effects of β-LAP on LPS-induced ROS production, which acts as a second messenger in inflammatory reactions and subsequent neuronal cell death [32, 33]. β-LAP significantly inhibited LPS-induced ROS production in the BV2 cells and primary microglia (Fig. 6a–c). Since NADPH oxidase is a major enzyme for microglial ROS release, we examined the effect of β-LAP on membrane (gp91^phox^, p22^phox^) and cytosolic (p47^phox^, p67^phox^) components of NADPH oxidase. β-LAP suppressed the expression and phosphorylation of p47^phox^ (Fig. 6d, e). Moreover, β-LAP inhibited the mRNA expression of gp91^phox^, without affecting p67^phox^ or p22^phox^ (Fig. 6d). We next examined the effects of β-LAP on HO-1 and NQO1, which mediate anti-inflammatory and anti-oxidant effects in the activated microglia [33, 34]. We observed that β-LAP increased LPS-induced HO-1 and NQO1 expression at mRNA and protein levels (Fig. 7a, b). Interestingly, we found that β-LAP itself also increased HO-1 and NQO1 expression.

### β-LAP activated Nrf2/ARE and PKA/CREB pathways

Because Nrf2 binds to ARE on the promoters of phase II anti-oxidant enzyme genes such as HO-1 and NQO1, and controls their expression [27, 34], we examined the effects of β-LAP on the binding of Nrf2 to ARE on HO-1/NQO1 promoters. We observed that LPS induced Nrf2 binding to ARE, which was enhanced by β-LAP (Fig. 7c). Moreover, β-LAP increased ARE-driven luciferase activity in the absence and presence of LPS (Fig. 7d). Previous studies of our own and by other groups reported that the PKA/CREB pathway contributes to the resolution of inflammation and ROS detoxification, and that PKA is an upstream modulator of HO-1 expression in microglia [35, 36]. In the present study, we observed that β-LAP itself increased the phosphorylation of CREB, which is a downstream target of PKA. In addition, β-LAP potentiated the CREB phosphorylation induced by LPS (Fig. 7e). β-LAP also increased the DNA binding and transcriptional activity of CREB in the absence or presence of LPS (Fig. 7f, g). Thus, the data suggest that the Nrf2/ARE and PKA pathways are largely involved in the anti-inflammatory/anti-oxidant mechanism of β-LAP, in association with other signaling pathways such as MAPKs, and PI3K/AKT.

## Discussion

Our present study demonstrates the anti-inflammatory properties of β-LAP in brain microglia and their underlying molecular mechanisms. β-LAP inhibited the expressions of iNOS and proinflammatory cytokines in LPS-stimulated microglia. In addition, β-LAP reduced the expression and activity of MMP-3, MMP-8, and MMP-9, which are inflammatory mediators in activated microglia [21–23]. By using a systemic inflammation mouse model, we confirmed the anti-inflammatory role of β-LAP. Thus, β-LAP inhibited microglial activation and the expression of proinflammatory molecules in the LPS-injected mouse brain. By mechanistic analysis, we showed that β-LAP inhibited the phosphorylation of MAPKs and AKT and the DNA binding activity of NF-κB/AP-1 induced by LPS. Furthermore, we found that β-LAP exerts anti-oxidant effects by reducing ROS production via suppression of NADPH oxidase subunit activity and/or expression, and upregulation of anti-oxidant enzymes such as HO-1 and NQO1. We showed that β-LAP activated Nrf2/ARE and PKA/CREB pathways, which are involved in the upregulation of HO-1/NQO1 expression. Therefore, β-LAP appears to act as an anti-inflammatory/anti-oxidant agent by modulating multiple signaling pathways (i.e., inhibition of MAPKs and PI3K/AKT, upregulation of Nrf2/ARE and PKA).

A previous study reported that β-LAP inhibits the mRNA expression of TLR4 signaling molecules in experimental autoimmune encephalomyelitis mice [37]. In the present study, however, β-LAP did not affect the mRNA expression of TLR4 or MyD88 in LPS-stimulated microglia (Additional file 1: Figure S1), suggesting that β-LAP exerts anti-inflammatory effects by modulating signaling downstream of TLR4/MyD88 and/or via a MyD88-independent pathway. In addition, we found that β-LAP also inhibited the inflammatory reactions induced by TLR2 or TLR3 agonists. Treatment with β-LAP inhibited NO and TNF-α production in BV2 cells stimulated with lipoteichoic acid (LTA; TLR2 agonist) or polyinosinic–polycytidylic acid (Poly I:C; TLR3 agonist) (Additional file 1: Figure S2). Thus, the data imply that the anti-inflammatory effect of β-LAP is not confined to TLR4 activation. Further studies are necessary to investigate the detailed mechanism underlying the effect of β-LAP on TLR signaling.

MMPs are zinc-dependent endopeptidases which are involved not only in normal brain development but also in various neuropathological conditions such as Alzheimer’s disease, Parkinson’s disease, stroke, and multiple sclerosis [38]. MMPs are aberrantly expressed in neuropathological conditions and cause breakdown of the blood–brain barrier (BBB), infiltration of peripheral immune cells, demyelination, and neuronal cell death [39, 40]. Our group recently reported that MMPs play an important role in various neuroinflammatory conditions [21–23]. We showed that MMP-3, MMP-8, and MMP-9 are upregulated in LPS or α-synuclein-stimulated microglia and mediate neuroinflammatory reactions. Thus, the specific inhibition of MMP-3, MMP-8, or MMP-9 suppresses iNOS and cytokine expressions in LPS or α-synuclein-stimulated microglia. We demonstrated that MMPs cleave the N-terminal extracellular domain of protease-activated receptor-1 and activate intracellular inflammatory signaling pathways in α-synuclein-activated microglia [21]. More recently, we showed that MMP-8 plays a pivotal role in neuroinflammation by activating TNF-α processing in microglia [22]. We also reported that TIMP-2, as an endogenous inhibitor of MMPs, has an anti-inflammatory effect by modulating MMP-3, MMP-8, and MMP-9 in activated microglia [29]. Based on these findings, our present study examined the effects of β-LAP on MMPs and TIMP-2 in LPS-stimulated microglia. We observed that β-LAP significantly suppressed the expression and activity of MMP-3, MMP-8, and MMP-9 with enhancement of TIMP-2 under in vitro and/or in vivo neuroinflammatory conditions, which may contribute to the anti-inflammatory properties of β-LAP.

In this study, we found that β-LAP induced phase II anti-oxidant enzymes such as HO-1 and NQO1, which are regulated through the Nrf2/ARE signaling pathway [34]. Under normal conditions, Nrf2 is sequestered by cytosolic Keap1, which serves as an adaptor to link Nrf2 to the ubiquitin ligase Cul3–Rbx1 complex that ubiquitinates and degrades Nrf2. However, upon stimulation by electrophilic agents or ROS, Nrf2 dissociates from its cytosolic docking protein Keap1, translocates into the nucleus, and binds to the ARE site [41]. It has been suggested that Nrf2 phosphorylation is involved in this release process. In the present study, we observed that β-LAP increased Nrf2 binding to ARE, as well as ARE-mediated transcriptional activity. However, we did not further examine the effect of β-LAP on the Nrf2 release mechanism related to Keap1; this would be an interesting study in the future.

It is well-known that β-LAP is a substrate and activator of NQO1, which catalyzes the oxidation of NADH to NAD^+^. A recent study reported that β-LAP induces HO-1 expression by increasing NQO1 activity and AMPK phosphorylation in RAW264.7 macrophages [18, 19]. As a mechanism to resolve the neurotoxic responses brought about by microglial activation, microglia usually express anti-inflammatory cytokines (i.e., IL-10, TGF-β1), suppressor of cytokine signaling (SOCS)-family proteins (i.e., SOCS1 and SOCS3), and anti-oxidant enzymes (i.e., HO-1, NQO1, SOD) [42, 43]. In the present study, we found that LPS itself increased HO-1 expression, which is associated with ARE-dependent transcription. We previously reported that the activation of PKA/CREB signaling is upstream of HO-1 expression and that the upregulation of the HO-1 and PKA pathway plays a key role in mediating the anti-inflammatory mechanism in LPS-stimulated microglia [35]. Therefore, the potentiation of HO-1/ARE and PKA/CREB by β-LAP may at least partly contribute to the anti-inflammatory and anti-oxidant effects of β-LAP in LPS-stimulated microglia. Interestingly, a recent study demonstrated that HO-1 knockout markedly increases MMP-9 expression in arteriovenous fistulas in mice and that MMP-9 induction reflects the prooxidant and proinflammatory effects recognized in a state of HO-1 deficiency [44]. Therefore, the upregulation of HO-1/NQO1 may at least partly contribute to the anti-inflammatory effects of β-LAP by suppressing proinflammatory molecules such as cytokines and MMPs in LPS-stimulated microglia.

A number of studies have reported therapeutic and health benefits of β-LAP supplementation in experimental animal models and clinical trials. The most extensively studied property of β-LAP is its anti-cancer potential, and β-LAP is currently being evaluated in clinical trials for treatment of cancer [9, 11]. In addition, β-LAP has beneficial effects on metabolic syndromes such as obesity, diabetes, hypertension, arterial restenosis, and salt-induced renal injury [45–48]. The anti-inflammatory/cytoprotective effects of β-LAP have also been reported in several disease models. β-LAP attenuates cisplatin-mediated acute kidney injury in mice by suppressing critical mediators for inflammation and ROS [15]. β-LAP has shown therapeutic effects against rheumatoid arthritis by inhibiting synoviocyte proliferation and suppressing MMP expression in chondrocytes [49]. β-LAP also protects against renal ischemia/reperfusion injury in mice by inducing NQO1 activation and subsequent inhibition of ROS [50]. In experimental autoimmune encephalomyelitis, an animal model of multiple sclerosis, the administration of β-LAP ameliorates the development of EAE by inhibiting the production of IL-12 family cytokines [37]. Interestingly, a recent study demonstrated that potentiation of NQO1 activity by feeding β-LAP prevents the age-dependent decline of motor and cognitive function in aged mice [51]. β-LAP also increases memory performance and prevents the loss of synapses in aged mice, suggesting the therapeutic potential of β-LAP for neurodegenerative diseases.

As to the BBB permeability of β-LAP, Huntingdon Life Sciences (UK) has reported that a minimal concentration of β-LAP penetrates into the rat brain, compared with other organs, under normal conditions (unpublished report). Therefore, we suggest two possibilities regarding the mechanism of β-LAP: first, β-LAP suppresses the peripheral inflammation induced by LPS and results in the inhibition of subsequent brain inflammation, and second, in systemic inflammatory conditions, BBB permeability is compromised and thus the penetration of β-LAP into the brain might be enhanced. In the latter case, β-LAP may directly modulate microglial activation. We believe that both of these mechanisms may be simultaneously involved in β-LAP action.

## Conclusions

The present study demonstrates that β-LAP inhibits neuroinflammation by modulating various inflammatory molecules and multiple signaling pathways. Considering the high demand for anti-inflammatory agents that can modulate microglial activation, β-LAP may be a promising preventive therapeutic agent against neuroinflammatory disorders such as Alzheimer’s disease and Parkinson’s disease.

## **Additional file**

Additional file 1:
**Figures S1 and S2. Figure S1.** Effects of β-lapachone on TLR4 and MyD88 expression in LPS-stimulated BV2 cells and mouse brain. **(A)** BV2 cells were pretreated with β-LAP (0.5, 1, and 2 μM) for 1 h, followed by treatment with LPS (100 ng/ml) for 6 h. Subsequently, total RNA was isolated and RT-PCR analysis was performed. **(B)** mRNA expression levels in the mouse brain were measured by RT-PCR analysis after 3 h of LPS injection. β-LAP (10 mg/kg, i.p.) was given daily for 4 days before LPS treatment. Representative gels of RT-PCR analysis are shown on the top, and quantifications of three independent experiments are shown in the bottom panel. Values correspond to the mean ± SEM of three independent experiments. **Figure S2.** Effects of β-lapachone on NO and TNF-α production in LTA (TLR2 agonist) or Poly I:C (TLR3 agonist)-stimulated BV2 microglial cells. Cells were incubated for 24 h with LTA (10 μg/ml) or Poly I:C (25 μg/ml) in the absence or presence of β-lapachone, and the amounts of NO and TNF-α were measured in the supernatants. The bars indicate the mean ± SEM of three independent experiments. **P* < 0.05, significantly different from stimulant-treated cells.

## Abbreviations

ARE: Anti-oxidant response element
BBB: Blood–brain barrier
CREB: cAMP response element-binding protein
CM: Conditioned media
EMSA: Electrophoretic mobility shift assay
ERK: Extracellular signal-regulated kinase
HO-1: Heme oxygenase-1
iNOS: Inducible nitric oxide synthase
β-LAP: β-Lapachone
LPS: Lipopolysaccharide
MAPK: Mitogen-activated protein kinase
JNK: c-Jun N-terminal kinase
MMP: Matrix metalloproteinase
NF-κB: Nuclear factor-κB
Nrf: Nuclear factor-E2-related factor
NQO1: NAD(P)H:quinone oxidoreductase 1
ROS: Reactive oxygen species
TIMP: Tissue inhibitor of metalloproteinase
