# Supplementary material for: β-Lapachone suppresses neuroinflammation by modulating the expression of cytokines and matrix metalloproteinases in activated microglia
Source: J Neuroinflammation. 2015 Jul 16;12:133. doi: 10.1186/s12974-015-0355-z (PMC4502557; doi:10.1186/s12974-015-0355-z)
Supplement: Additional file 1: — Figures S1 and S2. Figure S1. Effects of β-lapachone on TLR4 and MyD88 expression in LPS-stimulated BV2 cells and mouse brain. (A) BV2 cells were pretreated with β-LAP (0.5, 1, and 2 μM) for 1 h, followed by treatment with LPS (100 ng/ml) for 6 h. Subsequently, total RNA was isolated and RT-PCR analysis was performed. (B) mRNA expression levels in the mouse brain were measured by RT-PCR analysis after 3 h of LPS injection. β-LAP (10 mg/kg, i.p.) was given daily for 4 days before LPS treatment. Representative gels of RT-PCR analysis are shown on the top, and quantifications of three independent experiments are shown in the bottom panel. Values correspond to the mean ± SEM of three independent experiments. Figure S2. Effects of β-lapachone on NO and TNF-α production in LTA (TLR2 agonist) or Poly I:C (TLR3 agonist)-stimulated BV2 microglial cells. Cells were incubated for 24 h with LTA (10 μg/ml) or Poly I:C (25 μg/ml) in the absence or presence of β-lapachone, and the amounts of NO and TNF-α were measured in the supernatants. The bars indicate the mean ± SEM of three independent experiments. *P < 0.05, significantly different from stimulant-treated cells. [file 12974_2015_355_MOESM1_ESM.pdf]

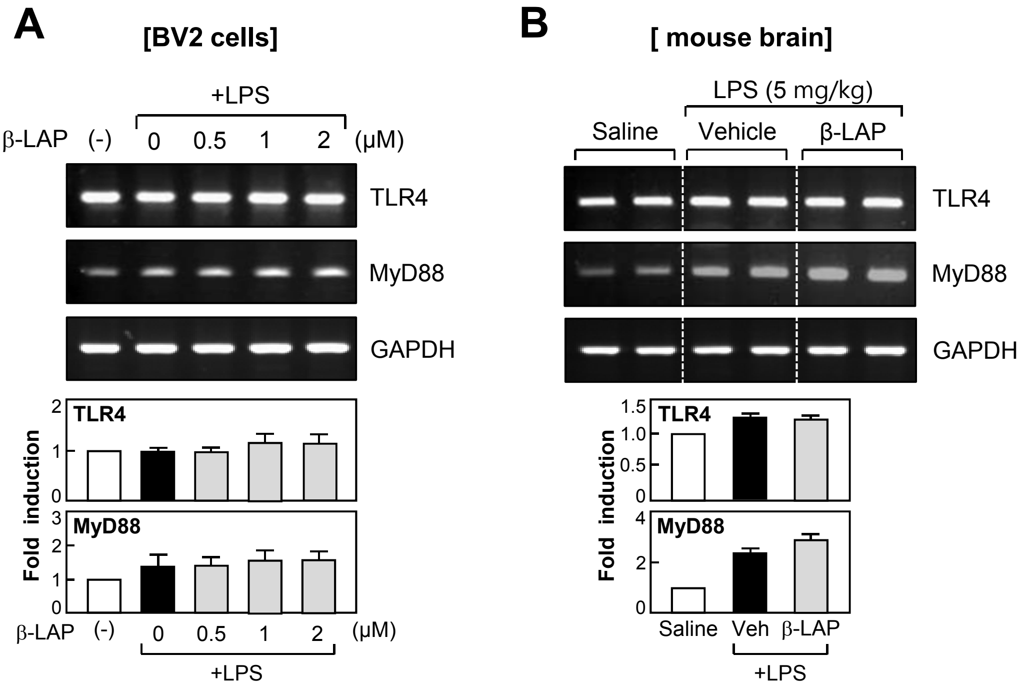

Supplementary Figure 1. Effects of β-lapachone on TLR4 and MyD88 expression in LPS-stimulated BV2 cells and mouse brain. (A) BV2 cells were pre-treated with β-LAP (0.5, 1, 2 μM) for 1 h, followed by treatment with LPS (100 ng/ml) for 6 h. Subsequently, total RNA was isolated and RT-PCR analysis was performed. **(B)** mRNA expression levels in the mouse brain were measured by RT-PCR analysis after 3 h of LPS injection. β-LAP (10 mg/kg, i.p.) was given daily for 4 days before LPS treatment. Representative gels of RT-PCR analysis are shown on the top, and quantifications of three independent experiments are shown in the bottom panel. Values correspond to the mean ± S.E.M. of three independent experiments.

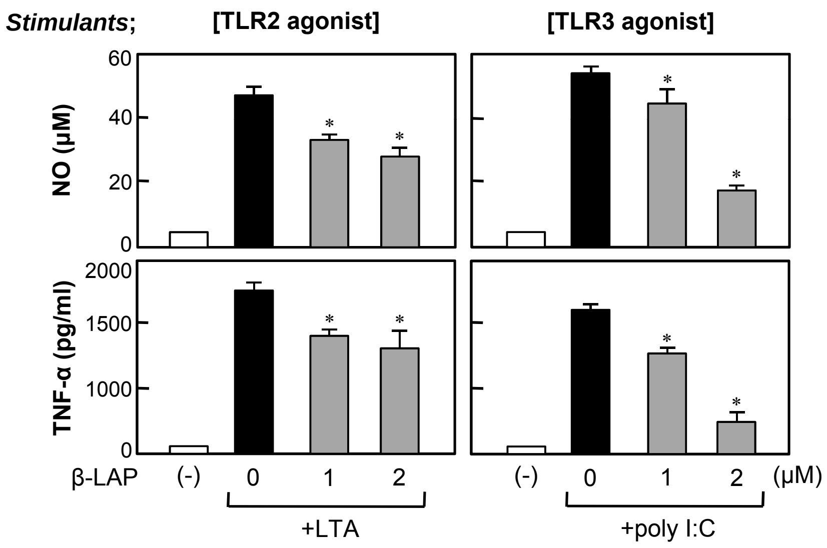

Supplementary Figure 2: **Effects of β-lapachone on NO and TNF-α production in LTA (TLR2 agonist) or Poly I:C (TLR3 agonist)-stimulated BV2 microglial cells.** Cells were incubated for 24 h with LTA (10 μg/ml) or Poly I:C (25 μg/ml) in the absence or presence of β-lapachone and the amounts of NO and TNF-α were measured in the supernatants. The bars indicate the mean ± S.E.M. of three independent experiments. *$P<0.05$; significantly different from stimulant-treated cells.
